# Disrupted functional connectivity during stroke recovery revealed via bedside optical neuroimaging

**DOI:** 10.1117/1.NPh.13.3.035002

**Published:** 2026-07-08

**Authors:** Karla M. Bergonzi, Broc A. Burke, Morgan Fogarty, Arefeh Sherafati, Tracy M. Burns-Yocum, Silvina L. Ferradal, Ben Julian A. Palanca, Rajat Dhar, Gyanendra Kumar, Jin-Moo Lee, Joseph P. Culver, Adam T. Eggebrecht

**Affiliations:** a L3Harris, Rochester, New York, United States; b University of Colorado Anschutz, Department of Anesthesiology, Aurora, Colorado, United States; c Washington University School of Medicine, Mallinckrodt Institute of Radiology, St. Louis, Missouri, United States; d St. Jude Children’s Research Hospital, Department of Psychology and Biobehavioral Sciences, Memphis, Tennessee, United States; e Evolytics | A Concord Company, Parkville, Missouri, United States; f Indiana University, Luddy School of Informatics, Computing, and Engineering, Department of Intelligent Systems Engineering, Bloomington, Indiana, United States; g Washington University School of Medicine, Department of Anesthesiology, St. Louis, Missouri, United States; h Washington University School of Medicine, Department of Neurology, St. Louis, Missouri, United States; i Mayo Clinic, Department of Neurology, Scottsdale, Arizona, United States

**Keywords:** functional imaging, neuroimaging, stroke recovery, diffuse optical tomography

## Abstract

**Significance:**

Current clinical care following ischemic stroke provides intermittent assessments, potentially missing early detection of neurological deterioration. Optical imaging offers the potential for a continuous, portable alternative to the current combination of physical exams and radiological imaging. However, optical imaging technology has lacked the crucial combination of portability, resolution, and coverage required to assess spatially distributed brain function.

**Aim:**

Here, we present a proof-of-principle study that applies recent advancements in portable high-density diffuse optical tomography (HD-DOT) instrumentation and a functional connectivity analysis strategy to assess spatially distributed brain connectivity. Point-of-care brain health assessments with these tools may inform clinical care and our understanding of brain injury throughout acute stages of stroke recovery.

**Approach:**

We measured spatially distributed cortical oxygenation with HD-DOT within the intensive care unit in N=13 patients with ischemic stroke within the first 72-h since last known normal. To assess brain function integrity, we developed a Similarity metric of functional connectivity (FC) through comparisons to FC in a healthy young adult population. To assess the sensitivity of this FC Similarity metric to disruptions caused by stroke, we compared these data with FC Similarity assessed in an older healthy cohort. We administered the NIH Stroke Scale to stroke patients to evaluate the potential of the FC Similarity metric to inform the severity of functional disruptions.

**Results:**

The FC Similarity metric applied to HD-DOT data in both acute stroke patients and healthy older controls exhibited a significant sensitivity to the presence of a stroke (Cohen’s d=1.5, $p=1.2\times 10^{-3}$) and a significant relationship with the degree of neurological disruption as measured by the NIH Stroke Scale ($R^{2}=0.69$, $p=4.7\times 10^{-4}$).

**Conclusions:**

We present a proof-of-principle for HD-DOT and the FC Similarity metric for assessing brain function during the acute stage of ischemic stroke recovery at the point-of-care.

## 1 Introduction

Brain injury evolves rapidly during the first 72 h after stroke onset, and therapeutic interventions aim to preserve viable brain tissue.^1^^–^^3^ Therefore, during this acute phase after stroke onset, early detection of neurological deterioration is essential. For critically ill stroke patients, the current standard of care consists of serial vital sign monitoring and neurological exams.^4^ Standard brain imaging methods, such as functional magnetic resonance imaging (fMRI) and positron emission tomography (PET), have demonstrated sensitivity to anatomical and functional disruptions due to stroke.^5^^,^^6^ However, these modalities do not support bedside monitoring of brain function. Therefore, novel approaches that enable early, continuous neurological assessment may improve our understanding of the acute phase of stroke recovery and inform clinical care.

Optical imaging, such as functional near-infrared spectroscopy (fNIRS), is promising for bedside neuroimaging due to the small console size and relative ease of use.^7^^–^^9^ Developments in high-density diffuse optical tomography (HD-DOT) have demonstrated dramatically improved image quality relative to sparse fNIRS systems and have been shown to produce fMRI-comparable maps of brain function.^10^^–^^14^ Importantly, HD-DOT can effectively map functional connectivity (FC) of spatially distributed resting state networks in adults^11^^,^^15^^–^^17^ and neonates.^12^^,^^18^^–^^21^

Here, we present a proof-of-principle study using portable HD-DOT hardware and an FC-based similarity metric to provide bedside assessments of disruptions in brain function during the acute phase of stroke recovery. Our results show that the FC Similarity is sensitive to functional disruptions caused by stroke. In addition, results show that the degree of disruption in brain function is significantly related to the degree of behavioral disruption, measured by the NIH Stroke Scale, a quantitative neurobehavioral clinical stroke measure acquired at the time of imaging. These findings represent a step toward establishing HD-DOT imaging and the FC Similarity metric as emerging tools for the clinical bedside assessment of brain function.

## 2 Materials and Methods

### 2.1 Participant Recruitment

The research was approved by the human research protection office at Washington University School of Medicine. We enrolled ischemic stroke patients who met the following criteria. Inclusion criteria included (1) age greater than 18 years, (2) anterior circulation ischemic stroke with onset within 72 h, and (3) National Institutes of Health Stroke Scale (NIHSS) greater than or equal to one at the time of initial evaluation in the ER. Exclusion criteria included (1) symptoms suggestive of a small subcortical stroke, (2) bilateral strokes, (3) prior stroke, (4) pregnancy, and (5) enrollment in an experimental therapeutic trial. Patients, or their legal representative, who met the inclusion and exclusion criteria were approached for consent. If the patient had difficulty with language production or comprehension due to the stroke, consent was obtained from the patient’s legally authorized representative.

Of the n=38 stroke patients enrolled, 25 were excluded from analyses: one due to hair incompatibility (thick dreadlocks), seven due to general excessive discomfort, five due to excessive movement, seven due to physician or family visits, two due to poor data quality caused by snoring, and three with less than 15 min of acquired data due to familial or clinical interruptions. We used a cutoff of 15 min of clean resting state data (see below for details) to optimize fidelity of the HD-DOT maps at the level of the individual participant.^22^^,^^23^ Of the remaining patients (n=13, four females, mean age=73.4±13.5  years), all had MCA strokes with four on the right side and nine on the left side. As patients were confined to a hospital bed, direct measures were not taken to prevent sleep. However, patients were monitored during data acquisition with detailed notes reporting movement and periods of sleep. Patient hair and skin characteristics were also recorded. This included hair thickness, length, and color, as well as skin tone (Fig. S1 in the Supplementary Material). A National Institutes of Health Stroke Scale (NIHSS) evaluation was performed within an hour of the HD-DOT scan.^24^

We recruited two control groups with an exclusion criterion of diagnosed neurological or psychological conditions. Young healthy volunteers (n=9) were enrolled with an inclusion criterion of age between 18 and 30 years. Of these nine subjects, seven are used in this study to generate an average young healthy reference baseline with two excluded due to motion artifacts limiting the total usable data to less than 15 min (n=7, mean age=25.9±1.7 years). Similarly, 11 older normal control subjects were recruited with the inclusion of age greater than 55 years (n=11, mean age=61.1±5.6 years). All healthy controls from both the young and older groups were imaged while lying supine on a hospital bed.

### 2.2 Clinical Structural Imaging

**Fig. 1:**
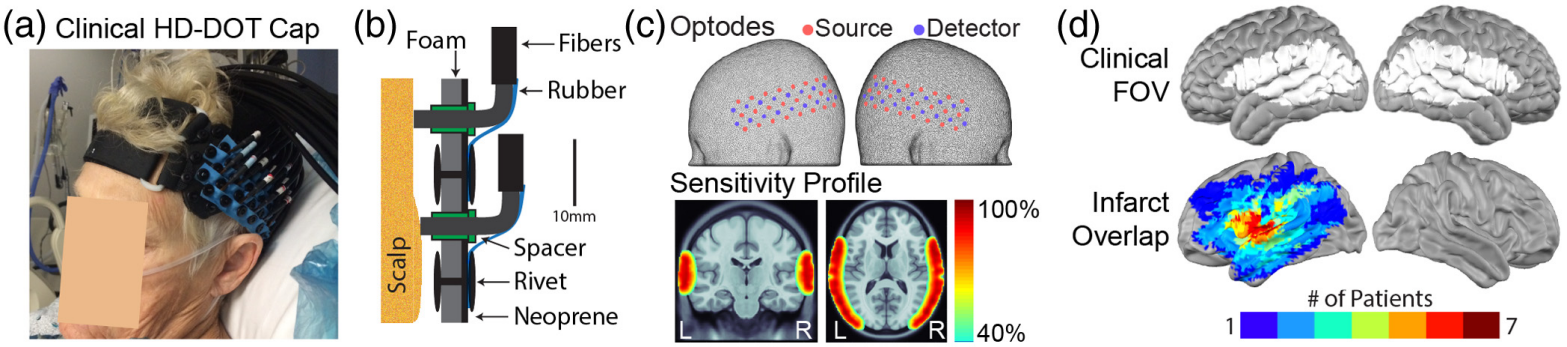
Clinical high-density DOT system. (a) The HD-DOT system images stroke patients at the bedside within the intensive care unit or their hospital room. The imaging cap straps around the forehead with the fibers traveling upward and crossing over the head to balance torque around the head to ensure a comfortable fit for the patient. The weight of the fibers is fully supported by the bed. (b) The cap consists of two semi-rigid, rubber-like patches of neoprene located over the MCA territory. A layer of foam neoprene on the inside of the cap adds stability to the spacers that guide the fibers through the cap. To keep the fibers pushed towards the center of the cap, rubber stripping (blue in panel a) sits tightly on the outside of the cap. Rivets hold the neoprene sheet, foam, and rubber stripping in place. (c) Interlaced grids of sources and detectors sit against the side of the head just above the ears. Overlapping measurements from source-detector separations of 1.3, 3.0, 3.9, and 4.7 cm provide uniform sensitivity. (d) The field of view of the cap, (white) covers bilateral aspects of pre-frontal, parietal, temporal, and occipital lobes. An infarct incidence map on an inflated view of the cortical ribbon for the seven patients with identifiable infarcts on clinical scans. For simplicity, all infarcts are shown here on the left-hand side.

**Fig. 2:**
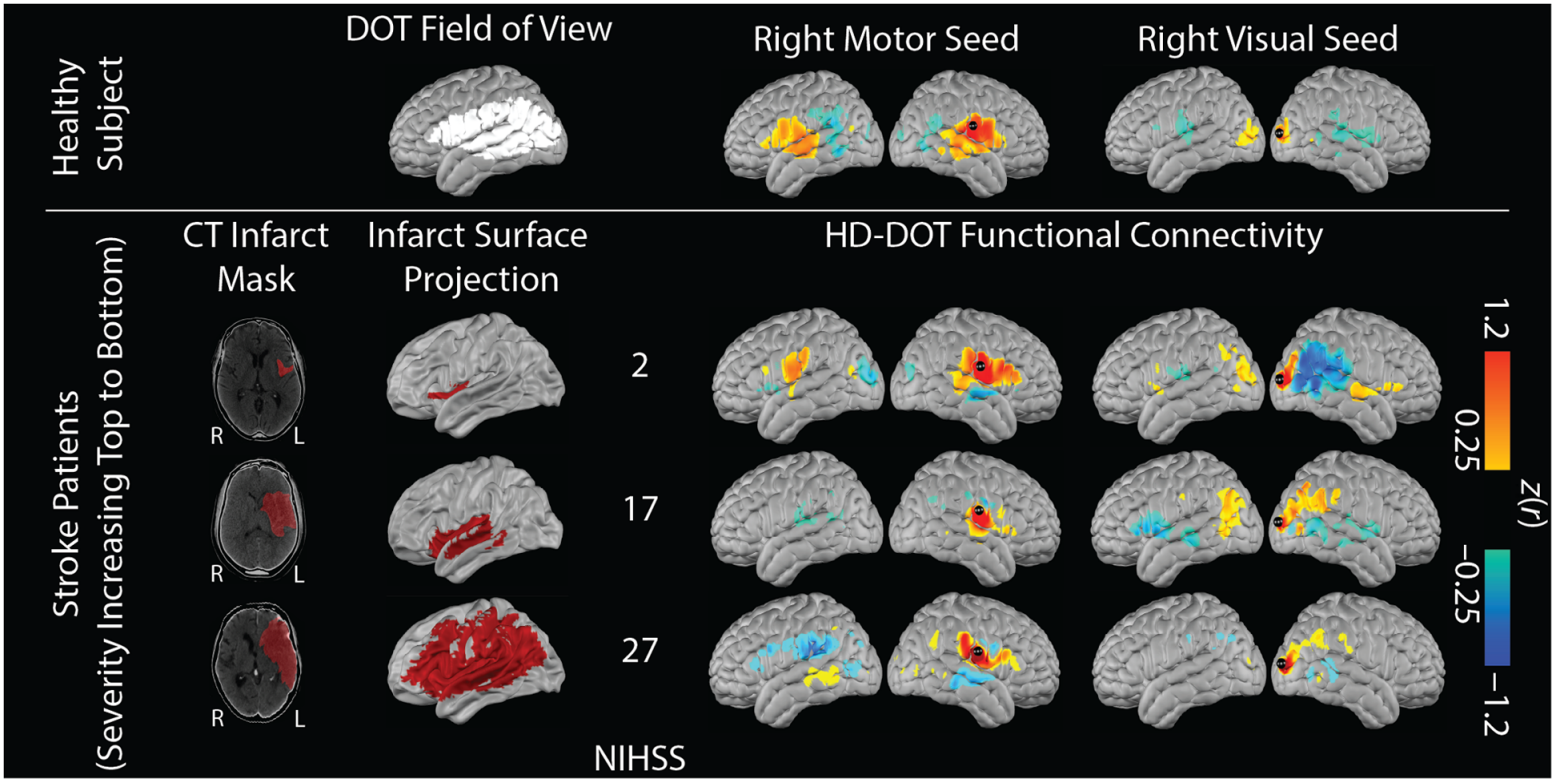
Bedside functional connectivity in acute stroke patients. Functional connectivity (FC) maps from a healthy subject show highly correlated regions around the region of interest (ROI) in addition to the bilateral region on the contralateral hemisphere. The FC maps of stroke patients for ROI placed in the right (unaffected in these examples) motor and visual cortices exhibit changes in connectivity patterns commensurate with injury. Patients with a low NIHSS have qualitatively similar bilateral patterns with high correlation values on both hemispheres whereas patients with more severe injuries show disrupted bilateral connectivity that is either close to zero or negative, indicating a loss of temporal synchrony between cortical regions of the same brain networks.

All evaluated stroke patients (n=38) underwent a head CT scan as part of their standard clinical care. The CT scans were performed during the 48 to 72 h following stroke onset using a Neurologica CereTom portable CT scanner (1.25×1.25×10  mm) or a Siemens Sensation Open scanner (0.5×0.5×3  mm) depending on patient stability and scanner availability. The CT scans were then evaluated by a board-certified neurologist to define a patient-specific ischemic infarct mask. Of the evaluated CT scans for included patients (n=13), masks were generated for nine of thirteen patients in the final analyses. Of the remaining four patients without visible infarct on CT, one patient underwent a brain MRI as part of standard clinical care (Siemens Symphony TIM, TRA Diffusion, 0.9×0.9×6  mm). This MRI was evaluated by the same neurologist as the CT scans, and an infarct mask was generated. The surface projections of these 10 masks were registered to the MNI atlas space and combined (right infarcts were reflected to the left-hand-side for visualization only) to generate an infarct incidence map [Fig. 1(d)]. Three sample infarct masks and surface visualizations are included in Fig. 2.

### 2.3 Clinical HD-DOT System

The clinical HD-DOT system was designed for high-fidelity bedside measurements without disrupting clinical care. The entire system fit within a custom-built hospital-compliant console (Minitec, New York) measuring 4 ft high, 3 ft long, and 2 ft wide. This small footprint enabled data collection in hospital rooms without disrupting clinical care. The clinical HD-DOT console supported bedside measurements from 48 sources and 34 detectors placed bilaterally in two rectangular grids on the participant’s head (Fig. 1). The continuous wave HD-DOT system contained LED sources, illuminating with near-infrared (NIR) wavelengths 750 and 850 nm (750-03 AU and OPE5T85, Roithner Lasertechnik), and avalanche photodiode (APD) detectors (Hamamatsu C5460-01). Source boxes (n=6) regulated power and delivered source encoding flashing patterns to 32 LEDs each (three 750 and one 850 nm LED per source position). The NIR light from the LEDs was directly coupled into 4-1 optical fiber bundles (CeramOptec, silicone cladded, 2.5-mm diameter bundles of 50  μm fiber, numerical aperture 0.66) via SMA connectors. Light collected from the head was transmitted to detector boxes (n=6, each housing up to six APD detectors) using similar 1-1 optical fibers and SMA connectors. The APDs were digitized by dedicated 24-bit analog-to-digital converters at 96 kHz (M32AD, RME).^25^ The system used temporal, frequency, and spatial encoding to sample the entire field of view at a 10 Hz framerate.^11^ Specifications for the light levels of the sources and noise levels of the detection opto-electronics have been previously described.^11^ To help ensure opto-electric stability, the system was turned on at least 30 min before use.

The imaging cap (Fig. 1) was designed with two central goals: (i) to provide stability for reliable optical coupling and (ii) to maximize ergonomic comfort for adult hospital patients. Custom right-angle optical fibers were coupled into a neoprene cap [Fig. 1(b)] that allowed the patient to recline in their bed. The imaging cap contained 48 sources and 34 detectors in two arrays with a first-neighbor separation of 1.3 cm [Fig. 1(c)], yielding up to 440 measurements per wavelength (750 and 850 nm). This arrangement produced a spatial resolution of ∼13  mm within 1.0 cm below the cortical surface [Fig. 1(c)].^10^^,^^26^ The field of view of this HD-DOT array encompassed bilateral aspects of temporal, occipital, parietal, and prefrontal cortices [Figs. 1(c) and 1(d)]. Real-time data quality assessment helped the study team optimize cap fit within 10 min, thereby maximizing data acquisition time (Fig. S2 in the Supplementary Material). See the Supplementary Material for details on cap fitting and real-time data quality assessment.

### 2.4 HD-DOT Data Processing and Reconstruction

Source-detector measurements with a standard deviation <7.5% of their mean signal were considered low-noise measurements and retained for further processing. The 10 Hz log-ratio source-detector measurements were band-pass filtered to 0.009 to 0.08 Hz. Superficial and systemic hemodynamic variance were removed through regression of the averaged low-noise first nearest-neighbor measurements.^27^ Low-noise measurement data were downsampled from 10 to 1 Hz and used for the image reconstruction.

Volumetric reconstructions of absorption coefficients at both 750 and 850 nm were obtained using Tikhonov and spatially variant regularization (with 0.1 coefficients for each), following well-established methods.^11^^,^^13^^,^^14^^,^^28^ Here, the sensitivity matrix was generated with an atlas representation of the adult head (Montreal Neurological Institute [MNI] 152-subject ICBM nonlinear registration atlas^29^^–^^31^). The adult atlas-based model of sensitivity was calculated using the NIRFAST light modeling toolbox,^32^ utilizing an FEM mesh based on the previously described head tissue segmentation and optical properties for scalp, skull, cerebrospinal fluid (CSF), gray, and white matter.^12^^,^^26^ Although some patients had head CT or MRI collected, these images did not provide the full head information needed for participant-specific head modeling. Thus, we used the same head atlas after adjusting for small changes in cap position and head size, as measured at the time of the scan, following previously described methods.^31^

Maps of relative concentration changes of oxyhemoglobin ($\Delta {\mathrm{HbO}}_{2}$) and deoxyhemoglobin (ΔHbR) were computed using the extinction coefficients of each hemoglobin species.^33^^,^^34^ Finally, the data were smoothed with a 12-mm FWHM Gaussian kernel and spatially registered to MNI atlas space. The field of view corresponds to the top 90% of the inverted sensitivity profile, relating to ∼2  cm below the head surface.^11^

### 2.5 Automated Motion Detection and Censoring

Noise from system fluctuations or patient motion can be misinterpreted as brain fluctuations, leading to erroneous functional connectivity results.^35^ Noisy measurements from system issues were identified as having a temporal standard deviation greater than 7.5% [Fig. S2(c) in the Supplementary Material] and were removed before image reconstruction. In addition, we utilized a time-point specific motion detection algorithm, termed global variance of the temporal derivatives (GVTD),^36^ which is analogous to the fMRI DVARS metric.^35^^,^^37^ The GVTD metric is calculated as the root mean square across the 13 mm measurements of the temporal derivative in log-ratio light levels. Temporal sections of data were classified as clean when they exhibited a GVTD value below an empirically defined threshold of 0.001 for at least 60 contiguous seconds. This fast and automated cropping of motion-contaminated data was performed before voxel-space reconstruction to maximize the sensitivity of the metric.^36^ Each epoch of measurement data remaining after automated motion detection was then preprocessed and reconstructed as previously published.^11^

### 2.6 Functional Connectivity Analyses

Each participant contributed 15 min of resting-state data after motion censoring. The global signal, averaged over the entire HD-DOT field of view in brain tissue, was regressed from every voxel.^27^ Region-of-interest (ROI) time traces were computed by averaging the voxels within a 5-mm radius sphere centered at a given ROI location, for example, motor and visual cortices (Fig. 2). ROI-based zero-lag functional connectivity (FC) maps were created by calculating the Pearson correlation coefficient between the ROI time trace and the time trace of every other voxel within the field of view (10,241 FC maps per subject with 10,241 brain voxels each). This is a common approach for FC analysis in both DOT and fNIRS.^16^^,^^11^^,^^38^^,^^39^ Correlation coefficients were Fisher-Z transformed [Eq. (1)] to convert the Pearson correlation coefficients (r) into a normally distributed variable (z) before further analysis,^11^ such that

$$z(r)=\mathrm{artanh}(r)=\frac{1}{2}\;\ln(\frac{1+r}{1-r}).$$ (1)

### 2.7 Statistical Analysis

To assess stroke-induced disruption of FC, we compared individual FC maps to reference datasets of healthy young adults. The FC Similarity metric for a given ROI location was calculated as the spatial Pearson correlation between the FC map of interest (e.g., stroke patient or older healthy participant) and the averaged young healthy reference population FC map. The collection of all Similarity values across all possible ROI in a given patient (or healthy control) create a distribution of FC Similarity values used for further analyses. To assess the effect of stroke on brain FC Similarity profiles, we used an independent samples t test to evaluate differences in patient-specific mean FC Similarity values between the stroke patients and the older healthy control group. To assess the relationship of disruption in resting state brain function with the severity of behavioral disruption, we calculated the Pearson correlation of the mean, standard deviation, kurtosis, and skewness of the similarity distributions of each patient with their NIHSS. To account for conducting four independent tests, we used the Bonferroni correction for multiple comparisons to adjust the thresholds for significance. Specifically, we considered the results as statistically significant if they passed a p value less than 0.05/4=0.0125. To evaluate the sensitivity to stroke severity, we related the skewness of the patient-specific FC Similarity distribution to the NIH Stroke Scale using regression analyses. Additional statistical analyses are detailed in the Supplementary Material.

## 3 Results

### 3.1 Bedside Mapping of Functional Connectivity with HD-DOT

The clinical HD-DOT system facilitated bedside imaging within the intensive care unit (ICU) during the initial 72-h acute phase of recovery from ischemic stroke (Fig. 1). An infarct incidence map of the 10 patients with identifiable infarcts on either clinical MRI or CT imaging shows damaged cortical tissue colocalized within the HD-DOT field of view [Fig. 1(d)]. To assess disruption in brain FC, we used a multi-ROI-based strategy (Fig. 2). Disruption in multiple FC maps is apparent at the individual patient level, as shown in three examples of stroke patients using ROI placement within right motor or right visual cortex. In the older control participant (Fig. 2, top), the FC maps exhibit strong interhemispheric correlation between homotopic counterparts for each of the right motor and right visual ROIs, indicating healthy FC. By contrast, in the stroke patients, the spatial map of correlation values shows clear disruptions, both contralateral and ipsilateral to the ROI. Further, the level of disruption increases along with stroke severity as indicated by the infarct volume (Fig. 2, left) and neurocognitive behavioral measures (NIHSS).

### 3.2 FC Similarity Metric Quantifies Disruption in Brain Function in Stroke Patients

**Fig. 3:**
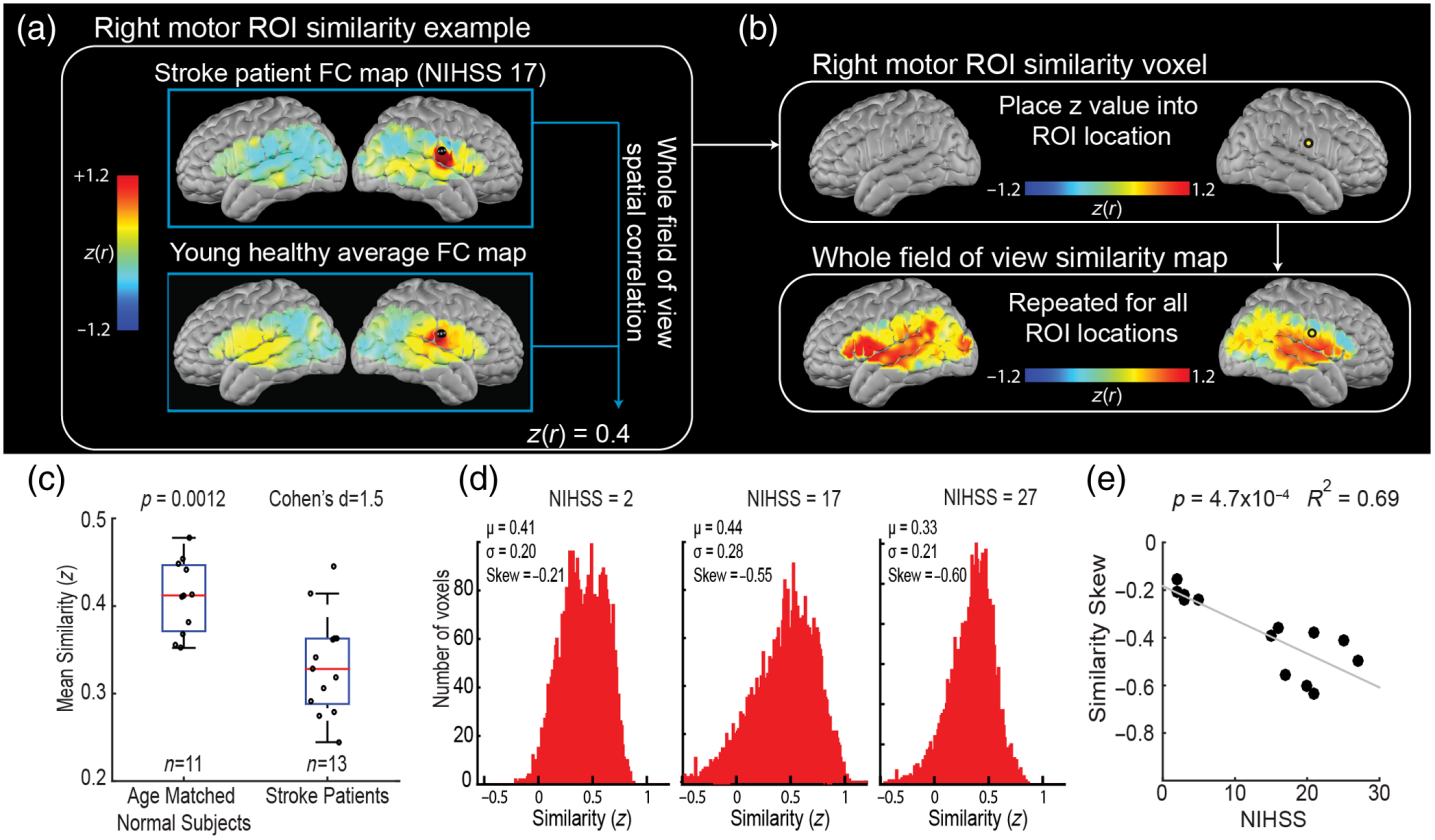
Similarity metric exhibits significant effects with the presence of stroke as well as with stroke severity. (a) A similarity value is calculated for an example right motor ROI based FC map between a stroke patient and the average young healthy population. The similarity metric is calculated as the spatial correlation between the two FC maps, resulting in a similarity value for this right motor ROI of z(r)=0.4. (b) A full distribution of similarity values was calculated at all possible ROI locations in the field of view (n=10,241) for each patient and participant. (c) Older healthy populations have greater mean similarity to young healthy subjects than the MCA stroke patients when comparing the per subject mean similarity (t=3.7, p=0.0012; Cohen’s-d=1.5). (d) As stroke severity increases, the distribution of similarity metrics across the field of view of the patient becomes more negatively skewed as shown in three representative subjects. (e) The skewness of the distribution of the similarity metric within each patient is significantly correlated with the NIHSS severity (n=13, $p<4.7\times 10^{-4}$, $R^{2}=0.69$).

To quantify disruption in resting state brain function, we calculated the FC Similarity metric as the spatial correlation between each ROI-based FC map from a stroke patient and the corresponding ROI-based FC map from a young healthy reference group [Figs. 3(a) and 3(b)]. To compare the group-level differences in mean Similarity value between the stroke patients and older healthy controls, we tested for differences in variance using Levene’s test, which was nonsignificant (p=0.363), and so equal variances were assumed. The mean Similarity values for stroke patients are significantly lower than the mean Similarity values for the older healthy control cohort (independent samples t test, t=3.7, $p<1.2\times 10^{-3}$). Notably, the group difference in mean Similarity value exhibits a strong effect size [Cohen’s-d=1.5, Fig. 3(c)].

### 3.3 Relationships of FC Similarity with Infarct Volume and Neurological Disruption

Given the observed spatially distributed disruption within FC maps of individual patients (Fig. 2), we investigated how the full distribution of Similarity values for a given participant may reveal distributed, heterogeneous levels of disruption in resting state brain activity [Fig. 3(d)]. The within-patient distribution of FC Similarity values reveals a remarkable increase in skewness magnitude toward stronger negative values as stroke severity increases. To assess the relationship between a behavioral measure of stroke severity and the level of disruption in brain FC, we calculated the Pearson correlation between the NIHSS and the skewness of the FC Similarity distribution of each stroke patient. The skewness of the FC Similarity values is strongly and significantly negatively correlated with the NIHSS given a Bonferroni-corrected p value threshold (p<0.0125) for multiple comparisons [n=13, r=−0.83, $R^{2}=0.69$, $p<4.7\times 10^{-4}$, Fig. 3(e)]. In contrast to skewness, we found low and insignificant correlation of the mean, standard deviation, and kurtosis FC Similarity distributions with NIHSS (Fig. S3 in the Supplementary Material). A strong positive relationship between the infarct volume and the NIHSS was quantified by Pearson correlation (n=10, r=0.713, $R^{2}=0.509$, p=0.031), whereas the Pearson correlation between the Similarity skewness and the infarct volume did not suggest collinearity (n=10, $R^{2}=0.030$, p=0.653).

## 4 Discussion and Conclusion

We demonstrate bedside mapping of brain FC within the first 72 h after ischemic stroke onset using HD-DOT. Although HD-DOT and fNIRS systems have been used clinically, they are often limited to neonates^12^^,^^20^^,^^40^ or provide only a small number of measurements.^8^^,^^9^ Notably, although this HD-DOT system is one of the largest reported adult clinical HD-DOT systems to date, the console did not disrupt standard clinical care within the neurological ICU. In addition, we show disruptions in FC maps measured at the bedside in acute stroke patients (Fig. 2) are significantly different in their FC Similarity to a healthy control population average than those from an older adult healthy control cohort [Fig. 3(c)]. Further, our results reveal a strong relationship between global disruption in resting state FC and a standard behavioral metric of neurological deficit [Fig. 3(e)].

Resting-state FC MRI provides an indicator of the brain’s physiological status in many neurological and psychiatric conditions.^41^^–^^44^ Prior fMRI studies have shown brain connectivity disruption in stroke during the postacute phase.^5^^,^^6^^,^^45^^–^^48^ However, the logistics of traditional brain imaging have hindered continuous neuroimaging during critical periods. We show that HD-DOT is well-suited for imaging during critical periods, as the system is portable while still covering important cortical regions including the MCA watershed (Fig. 1).

This adult clinical HD-DOT system contains 48 sources and 34 detectors that together provide up to 880 optical measurements within a 5-cm separation. Despite its portability, data quality remained consistent with that of previously reported systems, indicating the technical feasibility of bedside clinical imaging. In addition, real-time monitoring of multiple HD-DOT data-fidelity metrics helped preserve multiple datasets by enabling the user to track and correct poor scalp-fiber coupling (Fig. S2 in the Supplementary Material). These methods thereby minimize potential contamination of resting-state data, which is known to affect the fidelity of results.^35^^,^^36^^,^^49^^,^^50^ Examples of poor and good data quality, including light levels, temporal variance, light fall off, and signal-to-noise, demonstrate the direct impact of cap placement on data quality (Fig. S2 in the Supplementary Material).

To address challenges in quantifying FC disruption, we implemented an FC Similarity metric that is independent of infarct location and uses multiple FC maps that span cortical regions. The Similarity metric measures the spatial correlation of the spatially distributed pattern of a given ROI-based FC map of a stroke patient against a reference map of a healthy population average. This approach is similar to existing FC metrics that are used for connectome fingerprinting and subject identification in both fMRI and fNIRS.^41^^,^^51^^–^^54^ We found that stroke patients have lower mean Similarity values than the older healthy control cohort [Fig. 3(c)]. In evaluating correlation to NIHSS, we did not see a significant correlation between the mean, standard deviation, or kurtosis Similarity and the NIHSS (Fig. S3 in the Supplementary Material). However, we did find the skewness of the Similarity values to be significantly correlated with the NIHSS [Fig. 3(e)]. The negative skew indicates that the FC maps of stroke patients contain relatively more correlation values below the median, possibly due to local hemodynamic delays in the brain’s vascular supply.^55^

A primary limitation for optical neuroimaging is depth sensitivity. However, resting state networks have nodes within superficial cortex.^44^^,^^56^ Therefore, HD-DOT can detect deep brain lesions measured via disruptions in cortical-cortical node FC. Studies of chronic stroke using FC MRI have described disruptions in cortical-cortical connectivity due to deep subcortical infarcts.^46^^,^^47^^,^^46^ Another known limitation is the impact of hair and skin characteristics on optical neuroimaging signals.^57^ Here, patients with varying skin tones, hair lengths, hair thicknesses, and hair colors were included in the study (Fig. S1 in the Supplementary Material). This demonstrates the usability of the cap across varying phenotypes. However, one participant was excluded due to hair characteristics, highlighting the importance of further system development to ensure HD-DOT compatibility across populations. Recent advancements in fNIRS system designs have focused on overcoming these challenges.^58^

Future work will investigate real-time continuous monitoring over the first 72 h and beyond to examine how closely the Similarity metric correlates with the evolving NIHSS score. To further establish these methods, future studies with larger patient cohorts are required. Establishing the relationship between HD-DOT FC results, structural neuroimaging findings, and cognitive and behavioral outcome measures may provide novel optical imaging biomarkers of brain injury and adverse outcomes that will enable significant improvements in clinical care.

In summary, we present a proof-of-principle clinical bedside application of HD-DOT for assessing the disruption of FC during the acute stage of ischemic stroke. The magnitude of the disruption in brain function within a patient, as assessed with FC Similarity, is correlated with the degree of neurological dysfunction, as assessed with the NIHSS. These advances in data acquisition and analysis methods may provide tools to inform clinical care and deepen our understanding of evolving brain health across medical settings.

## Supplementary Material

10.1117/1.NPh.13.3.035002.s01

## Data Availability

The data presented in this article will be made publicly available on OpenNeuro following publication. Code for this article used NeuroDOT and can be made available upon request.
