# Supplementary material for: Disrupted functional connectivity during stroke recovery revealed via bedside optical neuroimaging
Source: Neurophotonics. 2026 Jul 8;13(3):035002. doi: 10.1117/1.NPh.13.3.035002 (PMC13341923; doi:10.1117/1.NPh.13.3.035002)
Supplement: Supplementary file 1 [file NPh_013_035002_SD001.pdf]

Supplemental Methods:

Clinical HD-DOT Imaging Cap

The neoprene cap provided both a locally rigid structure that maintained the regular HD grid, and flexibility that promoted fiber-scalp conformity for a wide variety of head shapes and sizes. Each of the 82 optical fiber tips was guided by a 'top-hat' style spacer to provide modest translation perpendicular to the head surface to facilitate consistent direct fiber-scalp coupling through hair. Elastic rubber strips on the outside of the cap, held in place with plastic rivets, maintained pressure on the right-angle tips to ensure adequate coupling of the optical fiber tip with the scalp (**Fig. 1b**) while also maintaining a ~3-5 mm penetration length of the optical fiber tip within the cap to ensure the tips comb through hair. The cap was attached to the patient using comfortable hook-and-loop straps across the forehead and over the top of the head. The imaging cap supported an HD imaging array by maintaining the 48 sources and 34 detectors in two interlaced rectangular arrays with first- through fourth-nearest neighbor separations as follows: 1.3, 3.0, 3.9, and 4.7 cm (**Fig. 1c**), with as many as 124, 170, 54, and 92 usable source-detector measurements per wavelength at the respective distances (up to 440 total measurements per wavelength). To maximize lateral coverage over the MCA watershed area, the grid was designed with left and right panels (each containing 24 sources and 17 detectors) that were symmetrically oriented to each other relative to the midline.

Fitting the HD-DOT Imaging Cap

To reliably acquire adequate coupling across the entire imaging cap, a simple set of steps was followed. First, the center of the cap was placed against the back of the head with the bottom row of fibers on the inion and angled such that the sides of the cap were situated approximately 0.5 cm above the helix-scalp intersect. Second, the cap was gently rubbed back and forth to comb the optical fiber tips through the patient's hair and to obtain stable coupling against the scalp for all 82 fiber tips. Third, two hook-and-loop straps were secured over the patient's eyebrows to secure the cap to the head. The weight of the fibers was supported by the bed around the patient to avoid discomfort. The position of the left and right pieces of the cap were checked for symmetric placement with the third source from the front placed just above the tragus at the dorsal-anterior ear-scalp connection. The cap fit procedure took approximately 10 minutes for each participant. Between participants, the cap was sanitized using alcohol wipes.

Real-time HD-DOT Data Quality Assessment

Real-time metrics of data quality were presented on a computer monitor on the HD-DOT console to facilitate efficient at-the-bedside optimization of cap coupling and position (**Fig. S1**). First, a schematic of the spatial layout of the optical coupling coefficients (proportional to the optical power attributed to each optode) for each source and detector position helped localize specific optical elements that were not optimally coupled (**Fig. S1a**). These fibers could be directly accessed by the user to improve combing through the hair and strengthen the coupling at the scalp interface. Next, source-detector measurement pairs passing a temporal noise-to-signal ratio (NSR) threshold of 7.5% or less were displayed as green lines on a similar spatial layout as the coupling coefficients (**Fig. S1b**). Third, a sliding window (5 second width) measurement of the

mean light level for each source-detector pair was displayed as a function of the source-detector separation (**Fig. S1c**). The quality of source-detector coupling was evaluated through inspection of the log-linear fall-off of the light-level plots as a function of source-detector distance. Proper coupling produces narrow variance in the light levels for a given source-detector distance. Poor coupling distorts the light-level to distance relationship by decreasing the light levels at short distances and increasing light levels at larger distances (due to optical cross-talk). Fourth, a histogram of the NSR for all measurement pairs was displayed (**Fig. S1c**). Optimal coupling of the HD-DOT array leads to values below 7.5% in the NSR histogram. A fifth real-time readout leveraged the pulsatility of the arterial blood flow, which is a reliable signature of strong coupling in a CW optical system [1]. The spatial layout of the pulse signature for each optode, calculated at the mean pulse band signal to noise of the given optode with its 30 mm measurement pairs (second nearest neighbor), was also displayed to aid in optimizing cap fit at the bedside (**Fig. S1d**). Displaying these metrics in real time helped the user optimize cap fit within 10 minutes, thereby maximizing data acquisition time for the study.

Statistical analysis

In addition to independent-samples t-tests comparing similarity values between stroke patients and the older healthy control group, we also tested for differences in the variance of similarity values between the groups using Levene's test. We assessed the effect size of the group difference in similarity values using Cohen's-d. To assess the relationship of disruption in resting state brain function with the severity of behavioral disruption, we calculated the Pearson correlation of the mean and the skewness of the similarity distributions of each patient with their NIHSS. For the nine patients with identifiable infarct on CT scan and one patient with infarct on MRI, we also calculated a Pearson correlation between the infarct volume and the NIHSS as well as between the infarct volume and the skewness of each patient's similarity distribution.

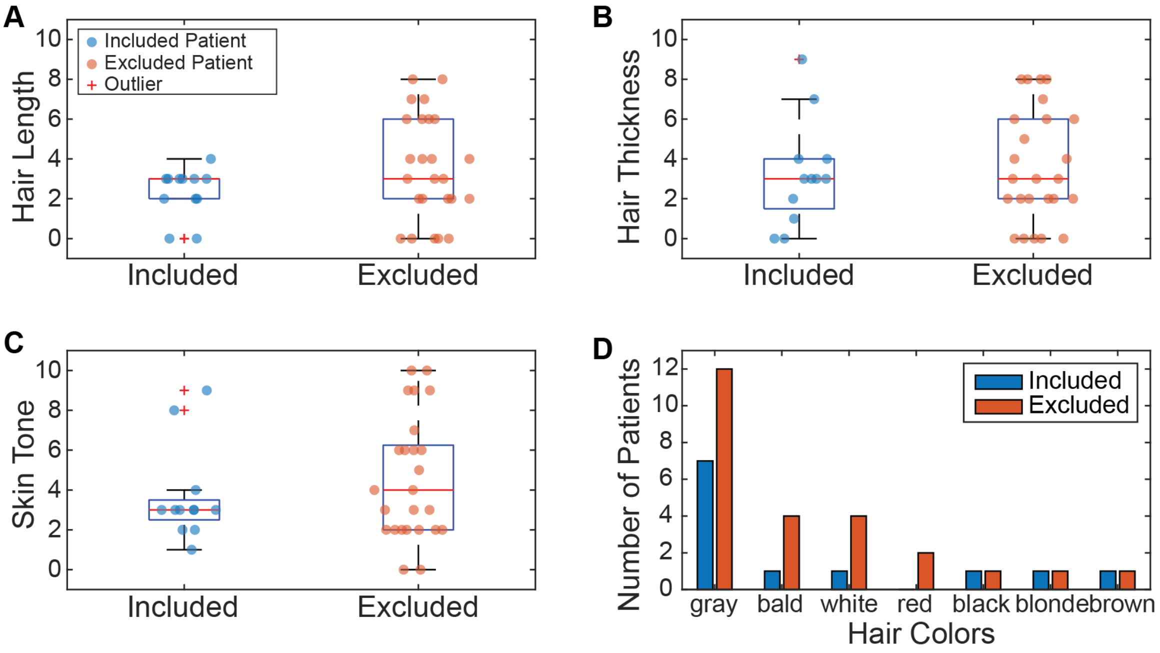

Fig. S1 | Hair and skin characteristics for stroke patients. As hair and skin properties can impact optical signals, we recorded hair length, thickness, and color as well as skin tone. Hair length **(A)** was rated from 0 (bald) to 10 (long hair), hair thickness (**B**) was rated from 0 (not thick) to 10 (very thick), and skin tone **(C)** was rated from 0 (fair) to 10 (dark). Box plots represent the median, 25th percentile, and 75th percentile across the included and excluded patient pools, with each point representing a single patient. Hair color **(D)** was reported for each patient, and most hair colors were represented in the included patient pool. Skin and hair characteristics are unavailable for one of the included participants.

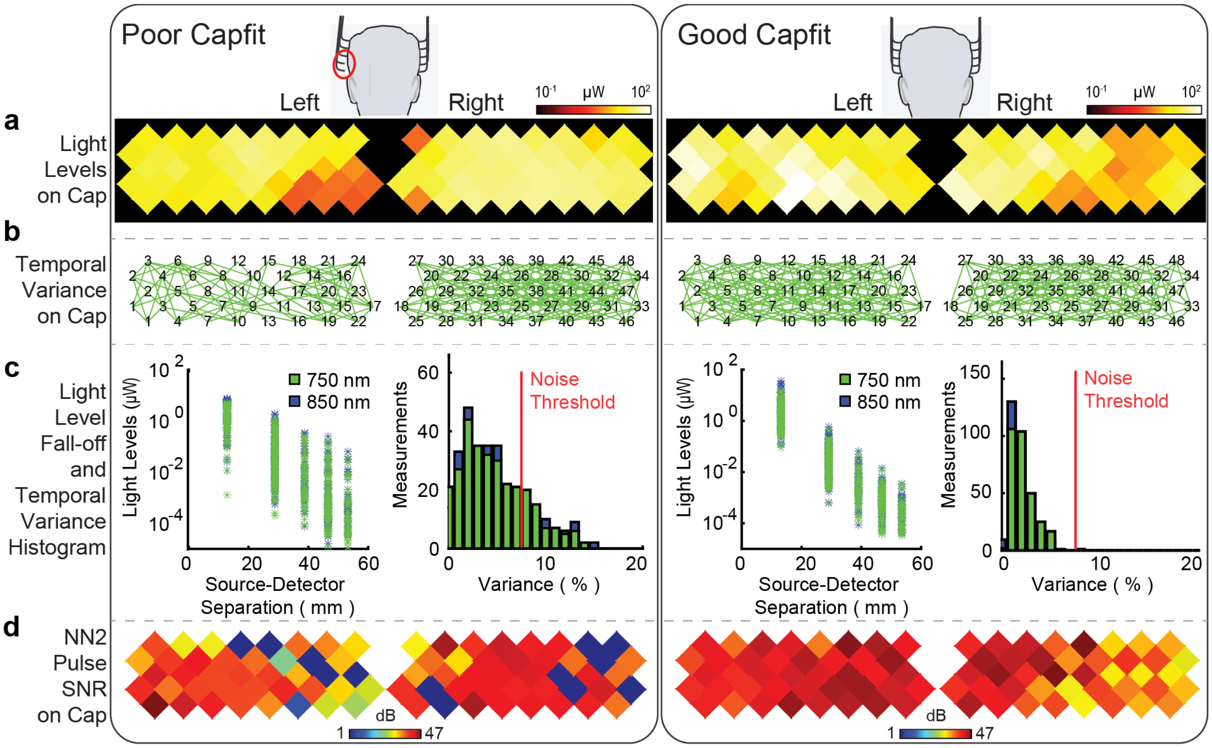

Fig. S2 | Real-time algorithms ensure adequate data quality at the bedside. a The spatial distribution of light levels for each source and detector is strongly affected by poor (left) or good (right) coupling of the fibers with the scalp. The red circle on the poor cap fit cartoon shows optodes sitting above the scalp which therefore have poor coupling. **b** Green lines reflect stable signals as poor coupling (left) introduces spurious and high temporal variance (>7.5% standard deviation in the signal level) that significantly and deleteriously affects data quality. A good cap fit (right) is apparent through the constant density of green lines. **c** Assessing the light fall-off as a function of distance (left sub-panels) as well as the histogram of measurement variance (right sub-panels) also provides guidance to optimize cap fit at the bedside. **d** Spatial distributions of the signal to noise (SNR) of the pulse component of the signal at each source/detector position (integrated over second nearest neighbor (NN2) pairs) provides a robust assay of cap fit and data quality. Low pulse SNR values are apparent with a bad cap fit (left) and can be improved by the user to provide a good cap fit (right).

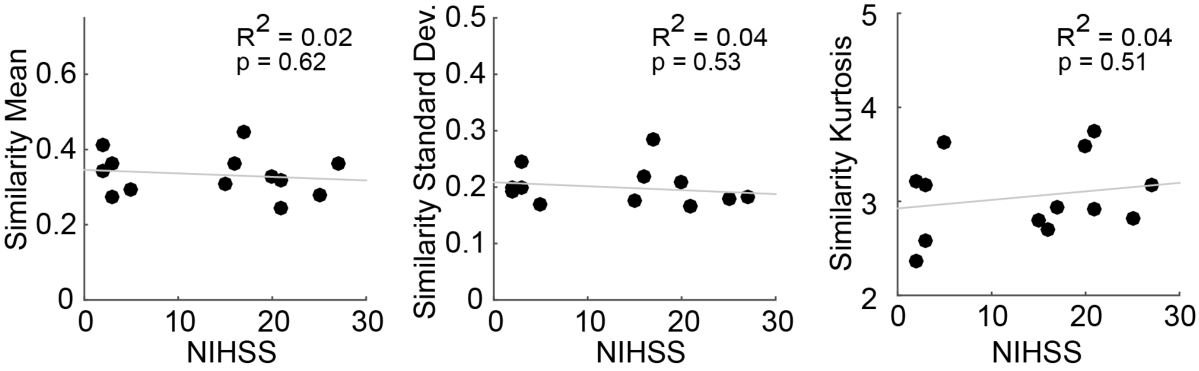

Fig. S3 | Similarity distribution properties with NIHSS. The mean (left), standard deviation (middle) and kurtosis (right) of the distribution of the similarity metric within each patient compared to the NIHSS severity demonstrates that mean, standard deviation, and kurtosis are not significantly correlated with stroke severity.

Supplemental References

1. Wheelock, M.D., J.P. Culver, and A.T. Eggebrecht, *High-density diffuse optical tomography for imaging human brain function.* Review of Scientific Instruments, 2019. **90**(5): p. 051101.
